# Extraordinary Case of Enlarged Spleen

**Published:** 1871-04

**Authors:** John G. Jay

**Affiliations:** Resident Student at the University of Maryland Hospital


### EXTRAORDINARY CASE OF ENLARGED SPLEEN

REPORTED BY JOHN G. JAY,

Resident Student at the University of Maryland Hospital.

The following remarkable case of enlargement of the spleen was admitted into the University Hospital, and attracted the attention of those who are connected with that institution. As such cases are comparatively rarely met with, even by those who have the most extended opportunities for observation, an account of the same may be of interest.

The patient, a negro man, aged twenty-eight years, by occupation a sailor, came into the house on the 28th day of April, 1870. From his account he was brought up in a malarious district, and had been employed for a number of years on some of the bay and river craft of the lower counties of the State of Maryland. During this period he had been very frequently the subject of bilious and intermittent fevers. According to his statement uneasy sensations referable to the spleen were first experienced about eighteen months previous to the date of his admission, and gradually increased in severity.

When he first came under my observation his condition was as follows: The abdomen was enormously distended by enlarged viscera and some accumulated fluid, dependent upon *mechanical causes.* The lower extremities were very edœmatous, pitting deeply upon pressure with the integument made tense from excessive dropsical effusion. When the case was admitted it was supposed to be one of Bright's disease of the kindey; but, upon further examination, the diagnosis of splenohæmia was clearly established. There was no puffiness of the eyelids nor swelling of the face and upper extremities; the countenance was not such as would warrant a belief in renal disease, and finally the absence of albumen in the urine seemed sufficient to exclude Bright's disease of the kidney. On the other hand the induration and tumefaction in the left hypochondriac region were plainly perceptible to palpation. With the assistance of percussion and manipulation through the skin and feeble abdominal walls, the outline of the enlarged spleen, with its peculiar fissures, could be distinctly defined. There had been frequent epistaxis, and also passages of blood from the bowels.

The case being diagnosed as one of enlarged spleen with the usual accompanying phenomena, the patient was put upon chalybeate tonics with quinine which failed, however, to give any good results, or to diminish in any way the enlarged organ.

A successful case of extirpation of the spleen, occurring in the practice of a German surgeon, having been reported in the English journals about the time of the admission of this patient into the hospital, the removal of this organ was suggested by Prof. Chisolm for the case in question, as it was apparent that internal medication would be of no avail. An opportunity, however, was not afforded to test the value of this operative expedient, as the death of the patient was induced through an accident which occurred to him one week after his admission into the wards. In moving along one of the hospital passages he fell down a short flight of steps, and in his distended condition was so seriously injured that he died within twenty-four hours.

After death an autopsy revealed the following condition: Before opening the body $7\frac{1}{2}$ quarts of dark bloody fluid were drawn off by paracentesis. The abdomen was then opened by a crucial incision ; the flaps being turned aside, the abdominal viscera were exposed in the following order: The left lobe of the liver extended about 3½ inches to the lelft of the median line, and overlapped the spleen to the extent of 4 inches. Its right lobe projected below the margin of the ribs 3½ inches. The weight of the liver was 7¾ pounds—the average weight of the healthy liver being from 3 to 5 pounds. The stomach was small and pushed over to the right side by the enlarged spleen. The omentum was much inflamed. The spleen extended from the pushed up diaphragm downward throughout the whole of the left side of the abdomen, reaching nearly to the pubes, also encroached on the right side for about 2½ inches beyond the median line. It was lobulated upon the right margin, the fissures being 4 in number, and from 1 to 2 inches in depth. This organ was adherent to the diaphragm.

There were also, attachments between other parts of the abdominal viscera. The weight of the spleen was 9¾ pounds; length, 16 inches; greatest breadth, 11½ inches. In contrast, the normal weight is 6 or 7 ounces. The heart was also some what hypertrophied and forced upward and out of position by the enlarged abdominal organs. The kidneys presented a normal appearance.

Although the size of this spleen was so very great, much larger than many put upon record, still larger ones have been mentioned by writers on pathological anatomy.

Several beautiful casts were taken of this spleen and placed in the Museum of the University of Maryland.

